# Multi-sequence MRI-based nomogram for prediction of human epidermal growth factor receptor 2 expression in breast cancer

**DOI:** 10.1016/j.heliyon.2025.e42398

**Published:** 2025-02-01

**Authors:** Mengyi Shen, Li Zhang, Dingyi Zhang, Xin He, Nian Liu, Xiaohua Huang

**Affiliations:** Department of Radiology, Affiliated Hospital of North Sichuan Medical College, Nanchong, China

**Keywords:** Breast cancer, Magnetic resonance imaging, Radiomics, Nomogram, Human epidermal growth factor receptor 2

## Abstract

**Objective:**

To develop a nomogram based on multi-sequence MRI (msMRI) radiomics features and imaging characteristics for predicting human epidermal growth factor receptor 2 (HER2) expression in breast cancer (BC).

**Methods:**

206 women diagnosed with invasive BC were retrospectively enrolled and randomly divided into a training set (n = 144) and a validation set (n = 62) at the ratio of 7 : 3. Tumor segmentation and feature extraction were performed on dynamic contrast-enhanced (DCE) MRI, T2-weighted imaging (T2WI), and apparent diffusion coefficient (ADC) map. Radiomics models were constructed using radiomics features and the radiomics score (Rad-score) was calculated. Rad-score and significant imaging characteristics were included in the multivariate analysis to establish the nomogram. The performance was mainly evaluated via the area under the receiver operating characteristic curve (AUC).

**Results:**

Edema types on T2WI (OR = 4.480, *P* = 0.008), enhancement type (OR = 7.550, *P* = 0.002), and Rad-score (OR = 5.906, *P* < 0.001) were independent imaging predictors for HER2 expression. Radiomics model based on msMRI (including DCE-MRI, T2WI, and ADC map) had AUCs of 0.936 and 0.880 in the training and validation sets, respectively, exceeding the AUCs of one sequence or dual sequences. With the combination of edema and enhancement types, the nomogram achieved the highest performance in the training set (AUC: 0.940) and validation set (AUC: 0.893).

**Conclusion:**

The developed multi-sequence MRI-based nomogram presents a promising tool for predicting HER2 expression, and is expected to improve the diagnosis and treatment of BC.

## 1 Introduction

Breast cancer (BC) is one of the most common malignant tumors in the world [1], seriously threatening women's health. About 10 %–15 % of BC cases show human epidermal growth factor receptor 2 (HER2) overexpression [2], leading to higher invasiveness and worse prognosis [3]. Besides, HER2 is one of the key indicators for the intrinsical classification of BC, and different classifications are closely related to treatment strategies [4]. Neoadjuvant therapy is the standard treatment for locally advanced or inoperable BC patients, and HER2 expression has been shown to influence the choice of therapy regimens [5]. The endocrine therapy is effective for part of HER2-negative patients, while the HER2 targeted therapy paired with chemotherapy is recommended for HER2-positive patients [5,6]. Choosing appropriate neoadjuvant therapy regimens based on HER2 status can improve preoperative tumor staging, facilitate tumor resection, and increase disease-free survival rates [3,7]. Notably, early identification of HER2 status is essential for classification diagnosis and personalized therapy of BC before surgury. While immunohistochemistry (IHC) is commonly employed to detect HER2, an IHC score of 2+ indicates ambiguous HER2 status, necessitating further testing via fluorescence in situ hybridization (FISH) [8]. The additional testing not only delays the timely diagnosis and treatment but also creates psychological and economic burdens for patients. Meanwhile, IHC and FISH may increase the risk of bleeding and infection due to the invasiveness [9]. Therefore, it is crucial to investigate more convenient and noninvasive diagnostic tools to aid clinicians in assessing HER2 expression in breast cancer.

Compared to other imaging technologies, magnetic resonance imaging (MRI) shows higher sensitivity and accuracy for BC detection [10,11]. Radiomics can automatically extract features from medical images, achieve quantitative analysis [12]. MRI-based radiomics has been proved to be feasible in predicting HER2 expression of BC [2,[13], [14], [15], [16]]. Dynamic contrast-enhanced (DCE) MRI could provide the information of morphology and blood perfusion simultaneously, but the radiomics model based on single DCE-MRI sequence had a poor diagnostic performance for distinguishing HER2 positivity from HER2 negativity [13,14]. T2-weighted imaging (T2WI) can provide good contrast resolution without injection of contrast agents. The radiomics model based on T2WI and DCE-MRI performed better than that based on one sequence, however, the performance seemed moderate [2,14]. The apparent diffusion coefficient (ADC) map obtained from diffusion-weighted imaging offers valuable microscopic insights into tumor cells [17]. A prior study demonstrated that the model based on radiomics signature from ADC and DCE-MRI was able to distinguish HER2 positivity from HER2 negativity in BC [15]. Selecting the appropriate MRI sequences to enhance the model's performance is crucial, and further research is needed to identify which sequences or combinations are the most significant contributors. Another study integrated clinical indicators into a nomogram based on radiomics of multiple sequences (including ADC, DCE-MRI, and T2WI), achieving commendable performance. This nomogram presents a promising tool for predicting HER2 expression in BC [16]. Nevertheless, the clinical indicators (Ki-67 and histological grade) included in that nomogram are derived through biopsy, which is an invasive approach.

In this study, we analyzed and compared the prediction performance of DCE-MRI, T2WI, and ADC sequences, and developed a nomogram using radiomics features from multi-sequence MRI (msMRI) and imaging characteristics for predicting HER2 expression of BC.

## 2 Materials and methods

### 2.1 Patients

**Fig. 1:**
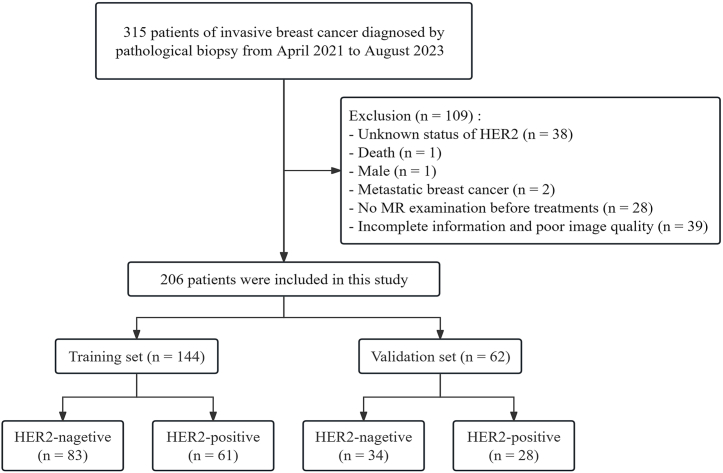
Flowchart of patient selection.

This retrospective study was approved by the Ethics Committee of our institution (NO. 2023ER163-1), and the informed consent was waived. 315 patients were enrolled from April 2021 to August 2023. The inclusion criteria included: (1) female patients with invasive BC diagnosed via pathological biopsy; (2) no treatment before the MR examination; (3) a definite HER2 pathological report. The exclusion criteria were: (1) metastatic BC; (2) poor image quality; (3) patients with incomplete clinical data. Finally, 206 patients (median age, 51 years) were included in this study (Fig. 1). Patients selected were divided into a training set (n = 144) and a validation set (n = 62) randomly at the ratio of 7 : 3.

### 2.2 MRI technique

The patients underwent breast scans using a 3.0T MR scanner (uMR790; Unite Imaging Healthcare, Shanghai, China) and a breast-phased array coil of 10 channels. Briefly, the patients were put in a prone position with bilateral breasts placed at the center of coil, and the scanning range included bilateral breasts and axillae. The scanning protocols and parameters were as follows: (1) axial fat-suppression T2-weighted imaging (FS-T2WI) with fast spin echo, (TR/TE, 5509/84; FOV, 340 × 340 mm; matrix size, 432 × 100 mm; slice thickness, 4 mm); (2) axial diffusion-weighted imaging (DWI) (TR/TE, 4000/66; FOV, 350 × 190 mm; matrix size, 192 × 100 mm; slice thickness, 4 mm; b = 0 and 800 s/mm2), and apparent diffusion coefficient (ADC) map derived from DWI; (3) axial fat-suppression dynamic contrast-enhanced T1-weighted imaging (DCE-T1WI) (TR/TE, 5.2/2.2; FOV, 340 × 340 mm; matrix size, 336 × 100 mm; slice thickness, 1 mm). The mask was scanned before the injection of contrast agents. Gd-DOTA (Hengrui Pharmaceuticals Co., Ltd, Jiangsu, China) was injected (0.2 mmol/kg, flow rate, 2.5 mL/s) through the median cubital vein using a high-pressure injector. Physiological saline (20 mL) was injected at the same rate, and continuous scanning was started after the injection. Each phase lasted for 69s (8 phases).

### 2.3 Clinical-imaging characteristics

Pathologists identified the substantial part of the tumor in combination with MRI, and then obtained HER2 status through core needle biopsy (CNB) under real-time guidance of ultrasound. The pathologic characteristics included lymph node metastasis, estrogen receptor (ER) status, progesterone receptor (PR) status, and Ki-67 index. The statuses of ER and PR were considered positive if at least 1 % invasive tumor cells were stained in the testing sample [18]. Based on the American Society of Clinical Oncology (ASCO)/College of American Pathologists (CAP), HER2-negative (HER2 [-]) was defined as IHC result of (0) or (1 +) while HER2-positive (HER2 [+]) was defined as IHC result of (3 +) [19,20]. FISH test was performed to clarify the HER2 status if the result was (2 +) in IHC (see Supplementary Fig. 1). Finally, 117 HER2 (−) and 89 HER2 (+) cases were enrolled. The molecular subtypes included luminal-A (n = 34), luminal-B HER2 (+) (n = 37), luminal-B HER2 (−) (n = 51), HER2-enriched (n = 53), and triple-negative (n = 31).

Reader 1 and reader 2 independently evaluated imaging characteristics, including: (1) maximum diameter was defined as the average value of measurements for three times on the largest cross-section obtained from DCE-MRI; (2) clinical tumor stage: tumor size ≤20 mm was defined as T1, 20 mm < tumor size ≤50 mm was defined as T2, tumor size >50 mm was defined as T3, T4 should be judged with the extent of invasion [21]; (3) edema types on T2WI were categorized into no edema, peritumoral edema, prepectoral edema, and subcutaneous edema (see Supplementary Fig. 2) [22]; (4) intratumoral hyperintensity on T2WI was defined as the watery hyperintensity on T2WI, without enhancement on all phases of DCE-MRI [23]; (5) time-signal intensity curve (TIC) was categorized into 3 patterns: persistent (Ⅰ), the signal intensity continuously increased; plateau (Ⅱ), the signal intensity was less variable and fluctuated within 10 % of the peak value; wash-out (Ⅲ), the signal intensity decreased over 10 % after reaching initial peak value [24]; (6) enhancement type was divided into mass enhancement and non-mass enhancement (NME) based on the 5th edition of the American College of Radiology Breast Imaging reporting and Data System (ACR BI-RADS) [25]. Any conflicts were solved by mutual discussion or reader 3.

### 2.4 Tumor segmentation and feature extraction

The peak enhancement in BC usually occurs in 60–120s after injection of contrast agents [26]. For DCE-MRI, we selected images of phase 2 to improve the accuracy of tumor segmentation. The images acquired from Picture Archiving and Communication System (PACS) were imported into 3D Slicer software (version 5.5.2) in a Digital Imaging and Communication in Medicine (DICOM) format. Two radiologists segmented the region of interest (ROI) of breast tumors without any knowledge of patients’ information using 3D Slicer. Segmentation was performed as follows: (1) each layer of the tumor in images was manually segmented; (2) only the lesion with the largest diameter was segmented if there were more than one mass; (3) segmentation was performed while avoiding necrosis, bleeding, and cystic changes around the tumor. Other MR sequences and different phases of DCE-MRI were taken into consideration when the ROI was segmented in one of the sequences. The voxel resolution of the images was resampled to 1 mm × 1 mm × 1 mm before extracting radiomics features to ensure the stability and repeatability of the features.

Reader 4 and reader 5 extracted radiomics features using the “*SlicerRadiomics*” plugin from 3D Slicer in the training set. Reader 4 segmented the ROI of all patients and extracted features, while reader 5 randomly selected one-third of the total number of patients, segmented the ROI, and extracted features. The consistency of the features was evaluated through intergroup correlation coefficients (ICC), and features with good consistency (ICC ≥0.75) were included in the next analysis. The data was standardized using the “*StandardScaler*” package after feature extraction to reduce the negative impacts on subsequent analyses. Homogeneity of variance was assessed via Levene's test. Univariate analysis (Student's *t*-test and Welch's *t*-test) was used to analyze data with equal variances and unequal variances respectively, and screen for features with statistical differences (*P* < 0.05). The selection of optimal features was achieved using the Least Absolute Shrinkage and Selection Operator (LASSO) algorithm with five-fold cross-validation under the criterion of minimum mean square error (MSE).

### 2.5 Model construction

**Fig. 2:**
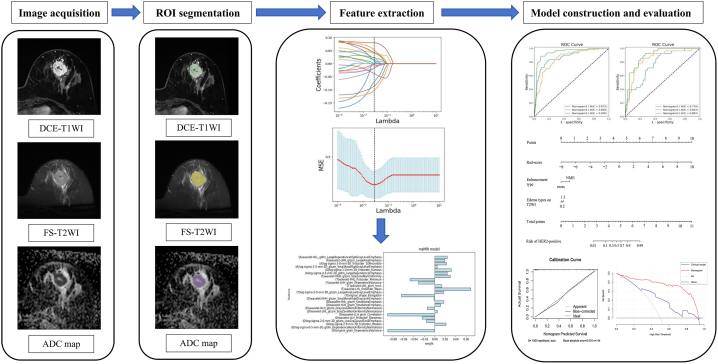
Radiomics workflow in this study.

Radiomics models were constructed by radiomics features, and the score of radiomics features (Rad-score) was calculated. The prediction model was constructed in the training set, while the validation set was used to validate the model. The clinical-imaging characteristics with statistical differences between HER2 (−) and HER2 (+) in the training set were incorporated into multivariate logistic regression analysis to select independent risk predictors and develop a clinical model. A nomogram was constructed based on Rad-score and independent risk predictors. The Logistic Regression (LR) classifier was used to construct all prediction models. The radiomics workflow was shown in Fig. 2.

### 2.6 Statistical analysis

Continuous variables’ normality was verified by Kolmogorov-Smirnov test. Independent sample *t*-test was employed for the comparison of continuous variables with normal distribution (mean ± SD), while the comparison of continuous variables with skewed distribution (median [interquartile range]) was approximated by Mann-Whitney *U* test. Classification variables were presented as the number of instances and group comparison was executed using chi-square or Fisher exact test. The correlation among the ordinal categorical variables were evaluated using Spearman correlation analysis.

The evaluation of model's predictive performance was achieved via the area under the curve (AUC) of receiver operating characteristic (ROC), sensitivity, specificity and accuracy. The optimal threshold of ROC curve was calculated using Youden index, and difference of AUC was assessed via Delong test. The fit degree was measured by the Hosmer-Lemeshow test and calibration curve, while the decision curve analysis (DCA) was used to estimate the clinical benefits. LR, Support Vector Machine (SVM), Random Forest (RF), Linear Discriminant Analysis (LDA), and XGBoost classifers with five-fold cross-validation resampling were used to demonstrate the model's generalisation ability.

Statistical analysis was executed on SPSS (version 25.0) and Python (version 3.10.9), significant differences were indicated statistically by *P* < 0.05.

## 3 Results

### 3.1 Clinical-imaging characteristics

The clinical-imaging characteristics in the HER2 (−) and HER2 (+) groups were shown in Supplementary Table 1. Table 1 provided the comparison of clinical-imaging characteristics between the training and validation sets. In the training set, significant statistical differences were observed in ER status (*P* = 0.001), PR status (*P* < 0.001), maximum diameter (*P* < 0.001), clinical tumor stage (*P* = 0.010), edema types on T2WI (*P* < 0.001), and enhancement type (*P* < 0.001). Notably, clinical tumor stage, edema types on T2WI, and enhancement type emerged as independent imaging predictors of HER2 expression in BC (Table 2). Consequently, a clinical model was established based on independent imaging predictors. The AUCs of the clinical model in the training set and validation set were 0.751 and 0.777, respectively (Table 3).

**Table 1:** Comparison of clinical-imaging characteristics between the training and validation sets.

Characteristics	Training set (n = 144)			Validation set (n = 62)		
	HER2 (−) (n = 83)	HER2 (+) (n = 61)	*P* value	HER2 (−) (n = 34)	HER2 (+) (n = 28)	*P* value
Age (years)	49.3 ± 9.9	51.0 (47.5, 56.5)	0.051∗	50.6 ± 10.5	53.8 ± 5.7	0.133
≤50	49 (59.0 %)	28 (45.9 %)	0.118	17 (50.0 %)	7 (25.0 %)	**0.044**
>50	34 (41.0 %)	33 (54.1 %)		17 (50.0 %)	21 (75.0 %)	
Menopausal status			0.061			0.100
Premenopausal	43 (51.8 %)	22 (36.1 %)		18 (52.9 %)	9 (32.1 %)	
Postmenopausal	40 (48.2 %)	39 (63.9 %)		16 (47.1 %)	19 (67.9 %)	
Lymph node metastasis			0.218			0.570
No	44 (53.0 %)	26 (42.6 %)		11 (32.4 %)	11 (39.3 %)	
Yes	39 (47.0 %)	35 (57.4 %)		23 (67.7 %)	17 (60.7 %)	
ER status			**0.001**			**< 0.001**
Negative	22 (26.5 %)	33 (54.1 %)		9 (26.5 %)	20 (71.4 %)	
Positive	61 (73.5 %)	28 (45.9 %)		25 (73.5 %)	8 (28.6 %)	
PR status			**< 0.001**			**< 0.001**
Negative	38 (45.8 %)	46 (75.4 %)		13 (38.2 %)	25 (89.3 %)	
Positive	45 (54.2 %)	15 (24.6 %)		21 (61.8 %)	3 (10.7 %)	
Ki-67 index			0.177			0.194
Low expression (≤20 %)	35 (42.2 %)	19 (31.2 %)		11 (32.4 %)	5 (17.9 %)	
High expression (>20 %)	48 (57.8 %)	42 (68.9 %)		23 (67.7 %)	23 (82.1 %)	
Maximum diameter (mm)	23.8 (19.2, 33.6)	31.7 (25.5, 43.5)	**< 0.001**∗	25.5 (19.1, 32.4)	39.6 ± 19.8	**0.004**∗
Clinical tumor stage			**0.010**∗∗			**0.007**∗∗
1	25 (30.1 %)	8 (13.1 %)		9 (26.5 %)	1 (3.6 %)	
2	52 (62.7 %)	45 (73.8 %)		22 (64.7 %)	19 (67.9 %)	
3	2 (2.4 %)	7 (11.5 %)		1 (2.9 %)	7 (25.0 %)	
4	4 (4.8 %)	1 (1.6 %)		2 (5.9 %)	1 (3.6 %)	
Intratumoral hyperintensity on T2WI			0.588			0.193
Absent	54 (65.1 %)	37 (60.7 %)		15 (44.1 %)	17 (60.7 %)	
Present	29 (34.9 %)	24 (39.3 %)		19 (55.9 %)	11 (39.3 %)	
Edema types on T2WI			**< 0.001**∗∗			**0.003**∗∗
No edema	65 (78.3 %)	22 (36.1 %)		22 (64.7 %)	6 (21.4 %)	
Peritumoral edema	8 (9.6 %)	12 (19.7 %)		5 (14.7 %)	11 (39.3 %)	
Prepectoral edema	0	7 (11.5 %)		0	1 (3.6 %)	
Subcutaneous edema	10 (12.1 %)	20 (32.8 %)		7 (20.6 %)	10 (35.7 %)	
TIC category			0.500			0.300∗∗
Ⅰ	8 (9.6 %)	5 (8.2 %)		4 (11.8 %)	1 (3.6 %)	
Ⅱ	45 (54.2 %)	39 (63.9 %)		22 (64.7 %)	16 (57.1 %)	
Ⅲ	30 (36.1 %)	17 (27.9 %)		8 (23.5 %)	11 (39.3 %)	
Enhancement type			**< 0.001**			**< 0.001**
Mass	76 (91.6 %)	40 (65.6 %)		31 (91.2 %)	13 (46.4 %)	
NME	7 (8.4 %)	21 (34.4 %)		3 (8.8 %)	15 (53.6 %)	

ER, estrogen receptor; PR, progesterone receptor; TIC, time-signal intensity curve; Ⅰ, persistent pattern; Ⅱ, plateau pattern; Ⅲ, wash-out pattern; NME, non-mass enhancement. ∗ and ∗∗ represented Mann-Whitney *U* test and Fisher exact test, respectively. *P* values less than 0.05 were bold.

**Table 2:** Multivariate logistic regression analysis of clinical-imaging characteristics in the training set.

Characteristics	Multivariate analysis		
	OR	95 % CI	*P* value
ER status	0.545	0.156–1.904	0.342
PR status	0.582	0.164–2.065	0.402
Maximum diameter	0.956	0.893–1.023	0.189
Clinical tumor stage	0.217	0.055–0.856	**0.029**
Edema types on T2WI	4.480	1.488–13.487	**0.008**
Enhancement type	7.550	2.140–26.642	**0.002**
Rad-score (msMRI)	5.906	3.299–10.574	**< 0.001**

ER, estrogen receptor; PR, progesterone receptor; OR, odds ratio; CI, confidence interval. *P* values less than 0.05 were bold.

**Table 3:** Predictive performance of models in the training and validation sets.

Model	Training set				
	AUC (95 % CI)	Sensitivity	Specificity	Accuracy	Threshold
DCE	0.842 (0.773–0.911)	0.705	0.855	0.782	0.566
T2WI	0.796 (0.719–0.872)	0.607	0.795	0.685	0.455
ADC	0.745 (0.662–0.828)	0.607	0.783	0.673	0.452
DCE + T2WI	0.880 (0.819–0.940)	0.770	0.831	0.770	0.525
DCE + ADC	0.867 (0.805–0.931)	0.738	0.843	0.776	0.536
T2WI + ADC	0.843 (0.775–0.911)	0.705	0.795	0.717	0.428
msMRI	0.936 (0.891–0.981)	0.869	0.904	0.869	0.531
Clinical	0.751 (0.669–0.834)	0.541	0.880	0.767	0.403
Nomogram	0.940 (0.896–0.983)	0.885	0.904	0.871	0.512

	Validation set				
	AUC (95 % CI)	Sensitivity	Specificity	Accuracy	Threshold

DCE	0.723 (0.593–0.852)	0.571	0.647	0.571	0.351
T2WI	0.702 (0.569–0.834)	0.607	0.794	0.708	0.534
ADC	0.729 (0.601–0.857)	0.500	0.853	0.737	0.460
DCE + T2WI	0.801 (0.688–0.915)	0.679	0.794	0.731	0.549
DCE + ADC	0.780 (0.662–0.899)	0.643	0.735	0.667	0.323
T2WI + ADC	0.838 (0.734–0.942)	0.607	0.853	0.773	0.450
msMRI	0.880 (0.790–0.971)	0.714	0.853	0.800	0.469
Clinical	0.777 (0.658–0.897)	0.536	0.706	0.600	0.331
Nomogram	0.893 (0.807–0.979)	0.714	0.824	0.769	0.380

CI, confidence interval.

### 3.2 Construction of radiomics model and nomogram

Three tasks were conducted to fully compare the radiomics models as follows. Task 1: Research on a single sequence. Task 2: Research on any two sequences. Task 3: Research on the combination of three sequences. 1223 radiomics features were extracted from the original images and images gone through Laplacian of Gaussian and wavelet transforms on DCE-T1WI, T2WI, and ADC respectively.

Task 1: First, 58, 321, and 791 features with ICC <0.75 were excluded from DCE-T1WI, T2WI, and ADC, respectively. Second, 804, 704, and 237 features with *P* > 0.05 were excluded via univariate analysis, respectively. Third, 350, 193, and 188 features were excluded via LASSO algorithm with five-fold cross-validation. Finally, only 11, 5, and 7 features (from DCE-T1WI, T2WI, and ADC, respectively) related to HER2 expression were screened. The DCE, T2WI, and ADC models were established based on the radiomics features.

Task 2: 2446 features of DCE + T2WI were obtained by combining the features of DCE and T2WI, Features of DCE + ADC and T2WI + ADC were obtained in the same way. First, 379, 849, and 1112 features with ICC <0.75 were excluded. Second, 1508, 1041, and 941 features with *P* > 0.05 were excluded via the univariate analysis. Third, 540, 536, and 380 features were excluded using LASSO algorithm. Finally, 19, 20, and 13 features were selected, respectively. The DCE + T2WI, DCE + ADC, and T2WI + ADC models were then established based on the radiomics features.

**Fig. 3:**
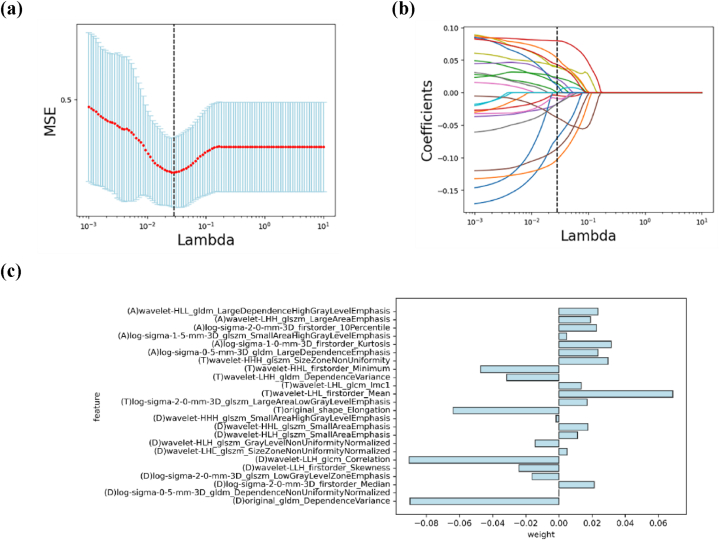
The procedure of dimensionality reduction using LASSO algorithm. The lambda (λ) value was selected under the minimum MSE criterion (indicated by the black dashed line) to screen the optimal features (a). Coefficients of radiomics features for different values of λ (b). Feature names and weights of the msMRI model after LASSO processing (c). A represented features from ADC map (n = 6); T represented features from FS-T2WI (n = 7); D represented features from DCE-T1WI (n = 11). GLCM_Correlation extracted through wavelet transform in DCE-T1WI acquired the highest absolute value of feature coefficients (0.090384).

Task 3: A total of 3669 features were obtained in the DCE + T2WI + ADC combination. This was consistent with the above steps, 1170, 1745, and 730 features were excluded via ICC, univariate analysis, and LASSO algorithm, respectively. Finally, 24 features were screened out and used for establishment of msMRI model (Fig. 3), and the interpretation of radiomics features was described in Supplementary Table 2.

The nomogram was constructed based on Rad-score of the model with best prediction performance and independent imaging predictors.

### 3.3 Evaluation of radiomics model and nomogram

The predictive performance of models was supplied in Table 3. The msMRI model achieved AUCs of 0.936 and 0.880 in the training and validation sets, respectively. In the training set, the AUC of msMRI model exceeded that of all other models evaluated in Task 1 and Task 2. Furthermore, the AUC of msMRI model in the validation set was higher than models in Task 1 and Task 2 (except for the T2WI + ADC model) (see Supplementary Fig. 3 and Table 3). The msMRI model showed the best performance, and therefore, we calculated its Rad-score (see Supplementary Table 4) and established a nomogram to further observe the performance.

**Fig. 4:**
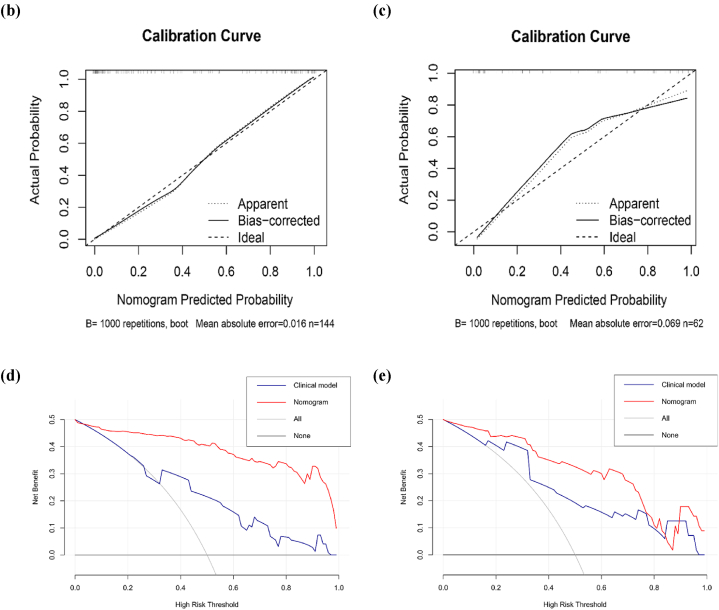
Performance of nomogram for predicting HER2 expression. Nomogram predicted the risk of HER2 positivity (a). The calibration curve for nomogram in the training set (b) and validation set (c). “Apparent”, “Bias-corrected”, and “Ideal” represented the entire cohort, the predictive performance of a nomogram corrected for bias through Bootstrapping (1000 repetitions), and a perfect prediction result, respectively. DCA for nomogram in the training set (d) and validation set (e).

**Fig. 5:**
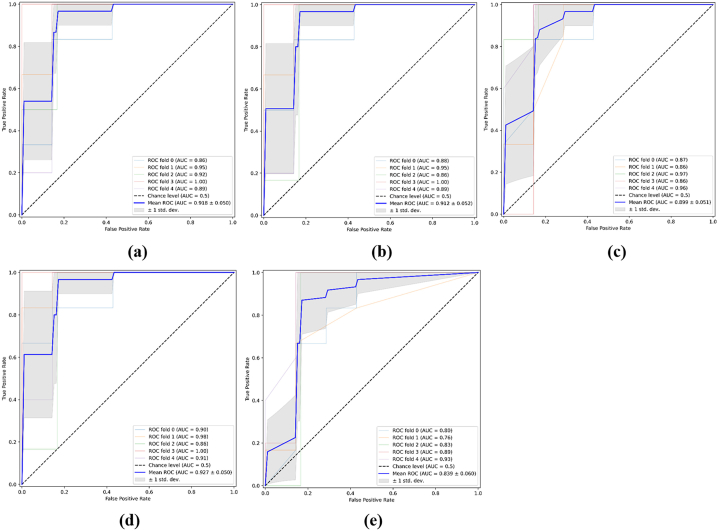
Logistic Regression (LR) (a), Support Vector Machine (SVM) (b), Random Forest (RF) (c), Linear Discriminant Analysis (LDA) (d), and XGBoost (e) classifers with five-fold cross-validation resampling were used to evaluate the generalisation ability of validation set in nomogram. The blue line represented the average AUC value obtained after five repeated samplings.

A nomogram was developed incorporating the Rad-score (msMRI), enhancement type, and edema types on T2WI (Fig. 4a). This model showed the highest AUCs of 0.940 in the training set and 0.893 in the validation set, outperforming the clinical model (*P* < 0.001 and *P* = 0.042, respectively). The Hosmer-Lemeshow test yielded P values of 0.25 and 0.41 for the training and validation sets, both exceeding 0.05. This indicated no significant discrepancy between the predicted and actual values, demonstrating a strong fit for the nomogram. Calibration curves were drawn to visualize the results of the Hosmer-Lemeshow test (Fig. 4b and c). DCA revealed that the nomogram provided greater clinical benefit compared to the clinical model when the risk threshold was greater than 5 % in the training set, and was between 5 % and 85 % in the validation set (Fig. 4d and e). The average AUC values for the nomogram's validation set in LR, SVM, RF, LDA, and XGBoost models with five-fold cross-validation resampling ranged from 0.839 to 0.927, indicating strong generalization capability of the nomogram (Fig. 5a–e).

## 4 Discussion

HER2 status is essential for classification diagnosis, precision treatment, and postoperative management of BC. This study evaluated the performance of radiomics models based on DCE-MRI, T2WI, and ADC sequences for predicting HER2 expression. The radiomics model derived from msMRI outperformed models based on single or dual sequences. The association between simple radiomics and clinical practice was not significant, therefore, we further included imaging characteristics to establish a visualized nomogram, providing quantitative imaging evidence for predicting HER2 expression. Edema and enhancement types displayed on MRI were independent risk predictors for HER2 expression. After combining imaging predictors, the nomogram achieved AUCs of 0.940 and 0.893 in the training and validation sets, respectively. Altogether, our results suggest that multi-sequence MRI-based nomogram is expected to become an important complementary method for clinical evaluation of HER2 expression in BC.

Radiomics has been proved to be a reliable tool for predicting HER2 expression in BC [[14], [15], [16]]. For instance, Zhou et al. [14] established a radiomics model based on radiomics signature extracted from DCE-T1WI and T2WI, achieving AUC values of 0.86 in the training set and 0.81 in the validation. Bian et al. [15] employed the radiomics model based on DCE-T1WI and ADC to distinguish HER2 (−) from HER2 (+), attaining AUCs of 0.793 and 0.778 in the training and validation sets, respectively. However, the performance of the models reported in previous studies was moderate, probably due to the selection of MRI sequences and inclusion of clinical predictors. The development of nomograms has improved the field of BC due to its potential to visualize and quantify features [[27], [28], [29]]. For instance, Xu et al. [16] developed a nomogram based on msMRI to predict HER2 status, achieving AUCs of 0.945 and 0.868 in the training and validation sets, respectively. Although it had relatively ideal performance, the clinical predictors included in that nomogram should be obtained through biopsy. The nomogram constructed in this study comprised noninvasive imaging characteristics and achieved comparable performance, demonstrating the robustness of MRI in predicting HER2 expression. Besides, we analyzed the efficiency of single sequence and sequence combinations. The performance of single-sequence and dual-sequence models was moderate, while the msMRI model had the best performance. Moreover, our findings indicated that the radiomics model utilizing both T2WI and ADC performed effectively. Previous research has investigated the role of unenhanced sequences in screening for BC and differentiating between benign and malignant breast tumors [11,30], highlighting their potential value. Moreover, the health risks posed to patients and the environmental concerns associated with gadolinium-based contrast agents cannot be overlooked [31,32]. Thus, it is crucial to explore the differentiation of HER2 expression using unenhanced sequences in our future study.

Wavelet features could capture the spatial heterogeneity of tumor tissue and provide a more nuanced understanding of the biological information encoded in the imaging data [33]. In this study, the majority of radiomics features extracted from msMRI were attributed to wavelet features (58.3 %), which suggested that wavelet features were closely related to BC, and BC was highly heterogeneous [13,34]. The key radiomics feature was GLCM_Correlation extracted from DCE-T1WI. Analysis of the similarity of elements in the row or column direction by Correlation revealed consistency in image texture. When the values of matrix elements were uniformly equal, the value of Correlation was larger, and vice versa. The value of Correlation for HER2 (+) (median value, 0.14) was less than that of HER2 (−) (median value, 0.15), *P* = 0.004, indicating that HER2 (+) had more uneven values of matrix elements and high heterogeneity.

Our study revealed that HER2-positive cases had a higher incidence of NME. This finding was consistent with the work of Ramtohul et al. [2] and Seyfettin et al. [35], who identified a significant correlation between HER2 positivity and NME. In MR images, NME exhibited a complex distribution and enhancement patterns, indicating a greater degree of invasiveness compared to mass enhancements characterized by smooth edges and limited extent. This aligned with the observed increased invasiveness in HER2-positive BC [36]. Meanwhile, a significant positive correlation between edema types on T2WI and HER2 (+) was implied (*r* = 0.406, *P* < 0.001), revealing that HER2 (+) patients were more likely to experience edema. The above findings suggested that clinicians should monitor NME and breast edema through DCE-MRI and T2WI, as they were related to the malignancy of breast tumors and HER2 expression of BC [[37], [38], [39]].

This study has some limitations. First, we employed various classifiers and cross-validation resampling to verify the robustness and generalization ability of the nomogram [16,40]. Future research should involve larger sample sizes and external validation to better reflect real-world conditions. Second, to avoid the interference of factors related to pathological types, we only included invasive BC, which was the most common pathological type. Third, the developed nomogram only included radiomics and imaging features, and future research should explore multidimensional clinical information related to HER2 expression. Furthermore, we analyzed two expression modes of HER2-positive and negative, however, HER2 low expression has an increasingly significance in BC [2,15].

## 5 Conclusions

Our results showed that the msMRI radiomics model outperformed those based on single or dual sequences. The multi-sequence MRI-based nomogram effectively predicted HER2 expression and demonstrated strong robustness and generalizability. This study presents a potential noninvasive method for predicting HER2 status, providing valuable insights for classification diagnosis, and treatment regimen selection in BC.

## CRediT authorship contribution statement

**Mengyi Shen:** Writing – review & editing, Writing – original draft, Formal analysis, Data curation, Conceptualization. **Li Zhang:** Writing – review & editing, Writing – original draft, Formal analysis, Data curation. **Dingyi Zhang:** Formal analysis, Data curation. **Xin He:** Formal analysis, Data curation. **Nian Liu:** Supervision, Formal analysis. **Xiaohua Huang:** Writing – review & editing, Methodology, Funding acquisition, Conceptualization.

## Ethical declaration

The institutional review board has granted approval for this retrospective study (2023ER163-1), and the requirement for informed consent has been waived.

## Data availability statement

Data will be available on request.

## Declaration of competing interest

The authors declare that they have no known competing financial interests or personal relationships that could have appeared to influence the work reported in this paper.

## References

[1] Arnold M., Morgan E., Rumgay H., Mafra A., Singh D., Laversanne M., Vignat J., Gralow J.R., Cardoso F., Siesling S., Soerjomataram I. (2022). Current and future burden of breast cancer: global statistics for 2020 and 2040. Breast.

[2] Ramtohul T., Djerroudi L., Lissavalid E., Nhy C., Redon L., Ikni L., Djelouah M., Journo G., Menet E., Cabel L., Malhaire C., Tardivon A. (2023). Multiparametric MRI and radiomics for the prediction of HER2-zero,-low, and-positive breast cancers. Radiology.

[3] Giffoni De Mello Morais Mata D., Chehade R., Hannouf M.B., Raphael J., Blanchette P., Al-Humiqani A., Ray M. (2023). Appraisal of systemic treatment strategies in early HER2-positive breast cancer—a literature review. Cancers.

[4] Khaled H., Gamal H., Lotayef M., Knauer M., Thürliman B. (2018). The St. Gallen international expert consensus conference on the primary therapy of early breast cancer 2017: Egyptian view. Breast Cancer Res. Treat..

[5] Cantini L., Trapani D., Guidi L., Bielo L.B., Scafetta R., Koziej M., Vidal L., Saini K.S., Curigliano G. (2023). Neoadjuvant therapy in hormone Receptor-Positive/HER2-Negative breast cancer. Cancer Treat Rev..

[6] Burstein H.J., Curigliano G., Loibl S., Dubsky P., Gnant M., Poortmans P., Colleoni M., Denkert C., Piccart-Gebhart M., Regan M., Senn H.J., Winer E.P., Thurlimann B. (2019). Estimating the benefits of therapy for early-stage breast cancer: the St. Gallen International Consensus Guidelines for the primary therapy of early breast cancer 2019. Ann. Oncol..

[7] Takada M., Toi M. (2020). Neoadjuvant treatment for HER2-positive breast cancer. Chin. Clin. Oncol..

[8] Harbeck N., Penault-Llorca F., Cortes J., Gnant M., Houssami N., Poortmans P., Ruddy K., Tsang J., Cardoso F. (2019). Breast cancer. Nat. Rev. Dis. Primers.

[9] Guo R., Lu G., Qin B., Fei B. (2018). Ultrasound imaging technologies for breast cancer detection and management: a review. Ultrasound Med. Biol..

[10] Chen Y., Wang L., Luo R., Liu H., Zhang Y., Wang D. (2023). Focal breast edema and breast edema score on T2-weighted images provides valuable biological information for invasive breast cancer. Insights Imaging.

[11] Rotili A., Pesapane F., Signorelli G., Penco S., Nicosia L., Bozzini A., Meneghetti L., Zanzottera C., Mannucci S., Bonanni B., Cassano E. (2023). An unenhanced breast MRI protocol based on diffusion-weighted imaging: a retrospective single-center study on high-risk population for breast cancer. Diagnostics.

[12] Rizzo S., Botta F., Raimondi S., Origgi D., Fanciullo C., Morganti A.G., Bellomi M. (2018). Radiomics: the facts and the challenges of image analysis. Eur. Radiol. Exp..

[13] Li C., Song L., Yin J. (2021). Intratumoral and peritumoral radiomics based on functional parametric maps from breast DCE-MRI for prediction of HER-2 and Ki-67 status. J. Magn. Reson. Imaging.

[14] Zhou J., Tan H., Li W., Liu Z., Wu Y., Bai Y., Fu F., Jia X., Feng A., Liu H., Wang M. (2021). Radiomics signatures based on multiparametric MRI for the preoperative prediction of the HER2 status of patients with breast cancer. Acad. Radiol..

[15] Bian X., Du S., Yue Z., Gao S., Zhao R., Huang G., Guo L., Peng C., Zhang L. (2023). Potential antihuman epidermal growth factor receptor 2 target therapy beneficiaries: the role of MRI-based radiomics in distinguishing human epidermal growth factor receptor 2-low status of breast cancer. J. Magn. Reson. Imaging.

[16] Xu A., Chu X., Zhang S., Zheng J., Shi D., Lv S., Li F., Weng X. (2022). Development and validation of a clinicoradiomic nomogram to assess the HER2 status of patients with invasive ductal carcinoma. BMC Cancer.

[17] Dai X., Shen Y., Gao Y., Huang G., Lin B., Liu Y. (2023). Correlation study between apparent diffusion coefficients and the prognostic factors in breast cancer. Clin. Radiol..

[18] Hammond M.E.H., Hayes D.F., Wolff A.C., Mangu P.B., Temin S. (2010). American society of clinical oncology/college of american pathologists guideline recommendations for immunohistochemical testing of estrogen and progesterone receptors in breast cancer. J. Oncol. Pract..

[19] Wolff A.C., Somerfield M.R., Dowsett M., Hammond M.E.H., Hayes D.F., Mcshane L.M., Saphner T.J., Spears P.A., Allison K.H. (2023). Human epidermal growth factor receptor 2 testing in breast cancer: ASCO–College of American Pathologists Guideline Update. J. Clin. Oncol..

[20] Wolff A.C., Hammond M.E.H., Allison K.H., Harvey B.E., Mangu P.B., Bartlett J.M., Bilous M., Ellis I.O., Fitzgibbons P., Hanna W., Jenkins R.B., Press M.F., Spears P.A., Vance G.H., Viale G., McShane L.M., Dowsett M. (2018). Human epidermal growth factor receptor 2 testing in breast cancer: American society of clinical oncology/college of American pathologists clinical practice guideline focused update. J. Clin. Oncol..

[21] Teichgraeber D.C., Guirguis M.S., Whitman G.J. (2021). Breast cancer staging: updates in the AJCC cancer staging manual, and current challenges for radiologists, from the AJR special series on cancer staging, AJR. Am. J. Roentgenol..

[22] Uematsu T. (2015). Focal breast edema associated with malignancy on T2-weighted images of breast MRI: peritumoral edema, prepectoral edema, and subcutaneous edema. Breast Cancer.

[23] Harada T.L., Uematsu T., Nakashima K., Sugino T., Nishimura S., Takahashi K., Hayashi T., Tadokoro Y., Watanabe J., Nakamoto S., Ito T. (2020). Is the presence of edema and necrosis on T2WI pretreatment breast MRI the key to predict pCR of triple negative breast cancer. Eur. Radiol..

[24] Linh L.T., Duc N.M., My T.T.T., Bang L.V., Thong P.M. (2021). Correlations between dynamic contrast-enhanced magnetic resonance imaging parameters and histopathologic factors in breast cancer. Clin. Ter..

[25] Spak D.A., Plaxco J., Santiago L., Dryden M., Dogan B. (2017). BI-RADS® fifth edition: a summary of changes, Diagn. Interv. Imaging.

[26] Chatterji M., Mercado C.L., Moy L. (2010). Optimizing 1.5-Tesla and 3-Tesla dynamic contrast-enhanced magnetic resonance imaging of the breasts. Magn. Reson. Imaging Clin..

[27] Zhang J., Wang G., Ren J., Yang Z., Li D., Cui Y., Yang X. (2022). Multiparametric MRI-based radiomics nomogram for preoperative prediction of lymphovascular invasion and clinical outcomes in patients with breast invasive ductal carcinoma. Eur. Radiol..

[28] Sang L., Liu Z., Huang C., Xu J., Wang H. (2024). Multiparametric MRI-based radiomics nomogram for predicting the hormone receptor status of HER2-positive breast cancer. Clin. Radiol..

[29] Wang X., Hua H., Han J., Zhong X., Liu J., Chen J. (2023). Evaluation of multiparametric MRI radiomics-based nomogram in prediction of response to neoadjuvant chemotherapy in breast cancer: a two-center study, clin. Breast Cancer.

[30] Kim K.W., Kuzmiak C.M., Kim Y.J., Seo J.Y., Jung H.K., Lee M.S. (2018). Diagnostic usefulness of combination of diffusion-weighted imaging and T2WI, including apparent diffusion coefficient in breast lesions: assessment of histologic grade. Acad. Radiol..

[31] Sharma P., Cheng J., Coulthard A. (2023). Where does the gadolinium go? A review into the excretion and retention of intravenous gadolinium. J. Med. Imaging Radiat. Oncol..

[32] Inoue K., Fukushi M., Sahoo S.K., Veerasamy N., Furukawa A., Soyama S., Sakata A., Isoda R., Taguchi Y., Hosokawa S., Sagara H., Natarajan T. (2022). Measurements and future projections of Gd-based contrast agents for MRI exams in wastewater treatment plants in the Tokyo metropolitan area. Mar. Pollut. Bull..

[33] Qin S., Lin Z., Liu N., Zheng Y., Jia Q., Huang X. (2023). Prediction of postoperative reintervention risk for uterine fibroids using clinical-imaging features and T2WI radiomics before high-intensity focused ultrasound ablation. Int. J. Hyperthermia.

[34] Zhang S., Wang X., Yang Z., Zhu Y., Zhao N., Li Y., He J., Sun H., Xie Z. (2022). Intra-and peritumoral radiomics model based on early DCE-MRI for preoperative prediction of molecular subtypes in invasive ductal breast carcinoma: a multitask machine learning study. Front. Oncol..

[35] Seyfettin A., Dede I., Hakverdi S., Asig B.D., Temiz M., Karazincir S. (2022). MR imaging properties of breast cancer molecular subtypes. Eur. Rev. Med. Pharmacol. Sci..

[36] Kubota K., Mori M., Fujioka T., Watanabe K., Ito Y. (2023). Magnetic resonance imaging diagnosis of non-mass enhancement of the breast. J. Med. Ultrason..

[37] Vasselli F., Fabi A., Ferranti F.R., Barba M., Botti C., Vidiri A., Tommasin S. (2022). How dual-energy contrast-enhanced spectral mammography can provide useful clinical information about prognostic factors in breast cancer patients: a systematic review of literature. Front. Oncol..

[38] Li Q., Huang Y., Xiao Q., Duan S., Wang S., Li J., Niu Q., Gu Y. (2022). Value of radiomics based on CE-MRI for predicting the efficacy of neoadjuvant chemotherapy in invasive breast cancer. Br. J. Radiol..

[39] Sung P., Lee J.Y., Cheun J.H., Choi I.S., Park J.H., Park J.H., Kim B.H., Oh S., Chu A.J., Hwang K.T. (2023). Prognostic implication of focal breast edema on preoperative breast magnetic resonance imaging in breast cancer patients. J. Breast Cancer.

[40] Ma C., Hui Q., Gao X., Xu D., Tang B., Pen M., Lui S., Chen X. (2021). The feasibility of dual-energy CT to predict the probability of symptomatic intracerebral haemorrhage after successful mechanical thrombectomy. Clin. Radiol..

