# Supplementary material for: Multi-sequence MRI-based nomogram for prediction of human epidermal growth factor receptor 2 expression in breast cancer
Source: Heliyon. 2025 Feb 1;11(3):e42398. doi: 10.1016/j.heliyon.2025.e42398 (PMC11847282; doi:10.1016/j.heliyon.2025.e42398)
Supplement: Multimedia component 1 [file mmc1.docx]

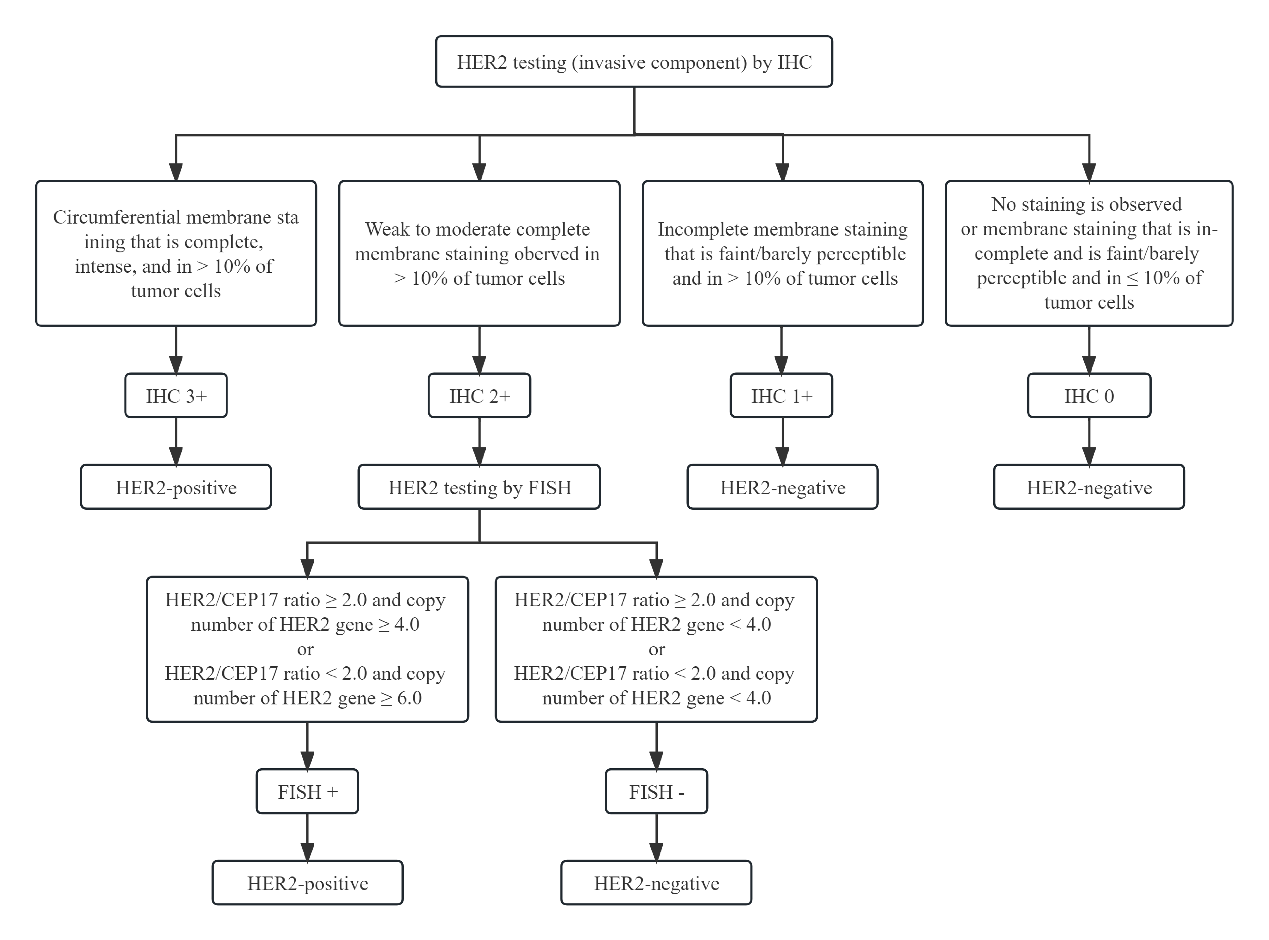


**Fig. S1.** Flowchart for detecting HER2 status through IHC and FISH. IHC, immunohistochemistry; FISH, fluorescence in situ hybridization; CEP17, chromosome 17 centromere.

**
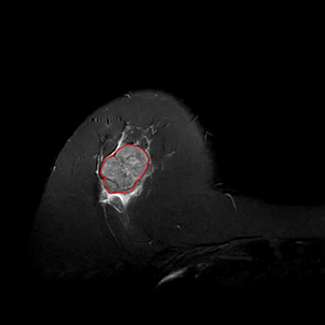

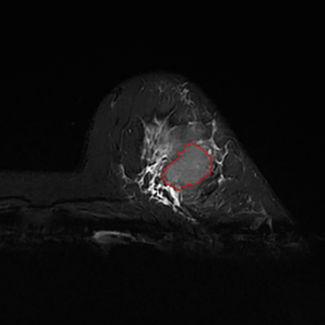

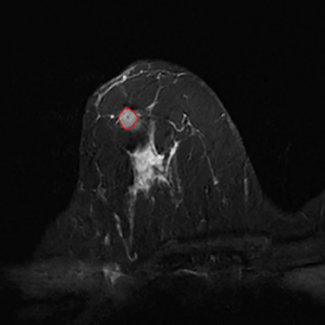

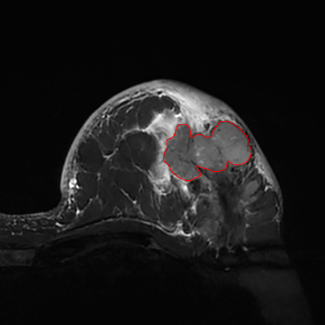
a b c d**

**Fig. S2.** Representative MR images of edema types on T2WI. The area delineated by the red line represented the tumor. No edema (score 0) (a); peritumoral edema (score 1), there was a watery high signal around the tumor, which did not extend to the pectoralis major muscle (b); prepectoral edema (score 2), the hyperintensity extended to the pectoralis major muscle, appearing linear or banded (c); subcutaneous edema (score 3), the range of hyperintensity expanded to the subcutaneous area (d).

**Table S1**

Comparison of clinical-imaging characteristics between HER2 (-) and HER2 (+).

| Characteristics | HER2 (-)  (n = 117) | HER2 (+)  (n = 89) | *P* value |
| --- | --- | --- | --- |
| Age (years) | 50.0 (44.0, 55.0) | 52.0 (49.0, 57.0) | **0.009**^*^ |
| ≤ 50 | 66 (56.4%) | 35 (39.3%) | **0.015** |
| > 50 | 51 (43.6%) | 54 (60.7%) |  |
| Menopausal status |  |  | **0.013** |
| Premenopausal | 61 (52.1%) | 31 (34.8%) |  |
| Postmenopausal | 56 (47.9%) | 58 (65.2%) |  |
| Lymph node metastasis |  |  | 0.437 |
| No | 55 (47.0%) | 37 (41.6%) |  |
| Yes | 62 (53.0%) | 52 (58.4%) |  |
| ER status |  |  | **< 0.001** |
| Negative | 31 (26.5%) | 53 (59.6%) |  |
| Positive | 86 (73.5%) | 36 (40.4%) |  |
| PR status |  |  | **< 0.001** |
| Negative | 51 (43.6%) | 71 (79.8%) |  |
| Positive | 66 (56.4%) | 18 (20.2%) |  |
| Ki-67 index |  |  | 0.064 |
| Low expression (≤ 20%) | 46 (39.3%) | 24 (27.0%) |  |
| High expression (> 20%) | 71 (60.7%) | 65 (73.0%) |  |
| Maximum diameter (mm) | 24.3 (19.2, 33.3) | 32.3 (25.0, 46.5) | **< 0.001**^*^ |
| Clinical tumor stage |  |  | **< 0.001**^**^ |
| 1 | 34 (29.1%) | 9 (10.1%) |  |
| 2 | 74 (63.3%) | 64 (71.9%) |  |
| 3 | 3 (2.6%) | 14 (15.7%) |  |
| 4 | 6 (5.1%) | 2 (2.3%) |  |
| Intratumoral hyperintensity on T2WI |  |  | 0.856 |
| Absent | 69 (59.0%) | 53 (60.2%) |  |
| Present | 48 (41.0%) | 35 (39.8%) |  |
| Edema types on T2WI |  |  | **< 0.001**^**^ |
| No edema | 87 (74.4%) | 28 (31.5%) |  |
| Peritumoral edema | 13 (11.1%) | 23 (25.8%) |  |
| Prepectoral edema | 0 | 8 (9.0%) |  |
| Subcutaneous edema | 17 (14.5%) | 30 (33.7%) |  |
| TIC category |  |  | 0.636 |
| Ⅰ | 12 (10.3%) | 6 (6.7%) |  |
| Ⅱ | 67 (57.3%) | 55 (61.8%) |  |
| Ⅲ | 38 (32.5%) | 28 (31.5%) |  |
| Enhancement type |  |  | **< 0.001** |
| Mass | 107 (91.5%) | 53 (59.6%) |  |
| NME | 10 (8.6%) | 36 (40.5%) |  |

ER, estrogen receptor; PR, progesterone receptor; TIC, time-signal intensity curve; Ⅰ, persistent pattern; Ⅱ, plateau pattern; Ⅲ, wash-out pattern; NME, non-mass enhancement. * and ** represented Mann-Whitney *U* test and Fisher exact test, respectively. *P* values less than 0.05 were bold.

**Table S2**

Interpretation of radiomics features in msMRI model.

| Sequence | Image | Category | Feature | Interpretation |
| --- | --- | --- | --- | --- |
| DCE | Wavelet-HHH | GLSZM | Small Area High Gray Level Emphasis | Record the number of occurrences of some kind of elements in the two-dimensional images |
|  | Wavelet-HHL | GLSZM | Small Area Emphasis |  |
|  | Wavelet-HLH | GLSZM | Small Area Emphasis |  |
|  |  |  | Gray Level Non-Uniformity Normalized | The variability of grayscale intensity values in images is measured |
|  | Wavelet-LHL | GLSZM | Size Zone Non-Uniformity Normalized | The variability in the volume of large and small regions is measured in images |
|  | Wavelet-LLH | GLCM | Correlation | Measure the similarity of elements in the row or column direction of the spatial GLCM, reflecting the consistency of image texture |
|  |  | First order | Skewness | Asymmetry of the distribution of values |
|  | LoG sigma 2.0 mm | GLSZM | Low Gray Level Zone Emphasis | Record the number of occurrences of some kind of elements in the two-dimensional images |
|  |  | First order | Median | The median value |
|  | LoG sigma 0.5 mm | GLDM | Dependence Non-Uniformity Normalized | The variability in the volume of large and small regions is measured in images |
|  | Original | GLDM | Dependence Variance | The spatial correlation properties of gray level are investigated to describe texture |
| T2WI | Wavelet-HHH | GLSZM | Size Zone Non-Uniformity | The variability in the volume of large and small regions is measured in images |
|  | Wavelet-HHL | First order | Minimum | The minimum value |
|  | Wavelet-LHH | GLDM | Dependence Variance | The spatial correlation properties of gray level are investigated to describe texture |
|  | Wavelet-LHL | GLCM | Imc1 | Informational measurement of correlation 1 |
|  |  | First order | Mean | The median value |
|  | LoG sigma 2.0 mm | GLSZM | Large Area Low Gray Level Emphasis | Record the number of occurrences of some kind of elements in the two-dimensional images |
|  | Original | Shape | Elongation | The relationship between the two largest principal components in the ROI |
| ADC | Wavelet-HLL | GLDM | Large Dependence High Gray Level Emphasis | The spatial correlation properties of gray level are investigated to describe texture |
|  | Wavelet-LHH | GLSZM | Large Area Emphasis | Record the number of occurrences of some kind of elements in the two-dimensional images |
|  | LoG sigma 2.0 mm | First order | 10 Percentile | Tenth percentile of values |
|  | LoG sigma 1.5 mm | GLSZM | Small Area High Gray Level Emphasis | Record the number of occurrences of some kind of elements in the two-dimensional images |
|  | LoG sigma 1.0 mm | First order | Kurtosis | Measurement of peak value in ROI |
|  | LoG sigma 0.5 mm | GLDM | Large Dependence Emphasis | The spatial correlation properties of gray level are investigated to describe texture |

GLSZM, Gray Level Size Zone Matrix; GLCM, Gray Level Coordination Matrix; LoG, Laplacian of Gaussian; GLDM, Gray Level Dependence Matrix.


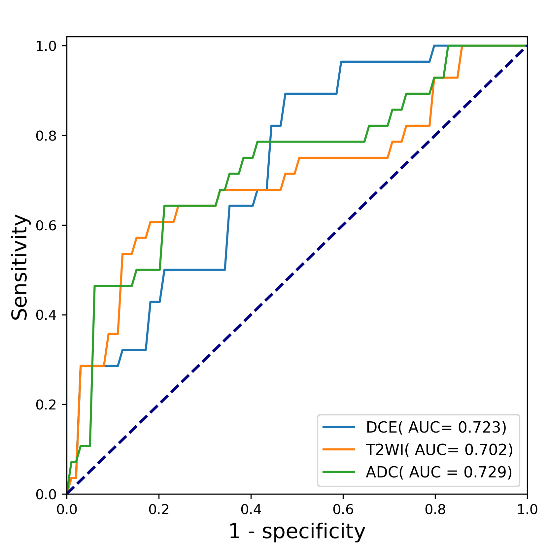

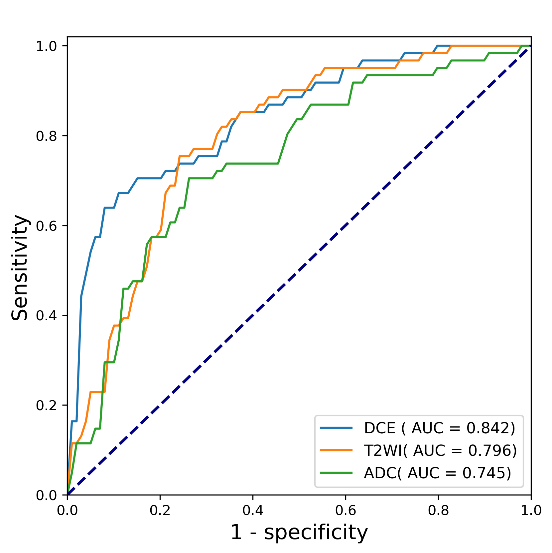


**(a) (b)**

**
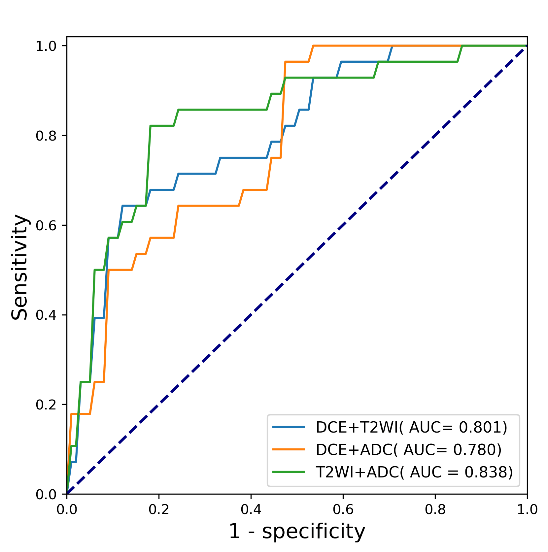

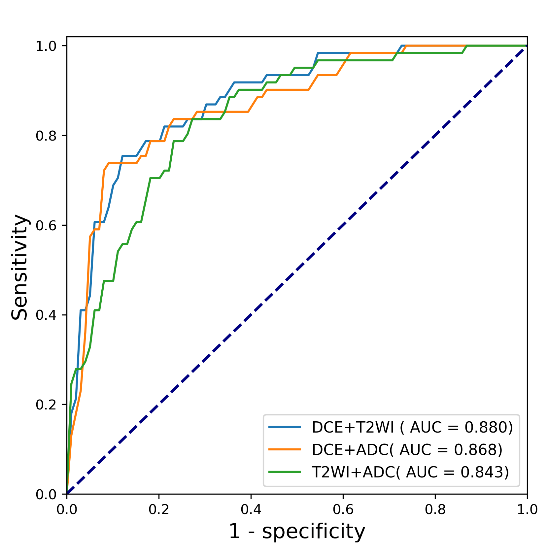
**

**(c) (d)**

**
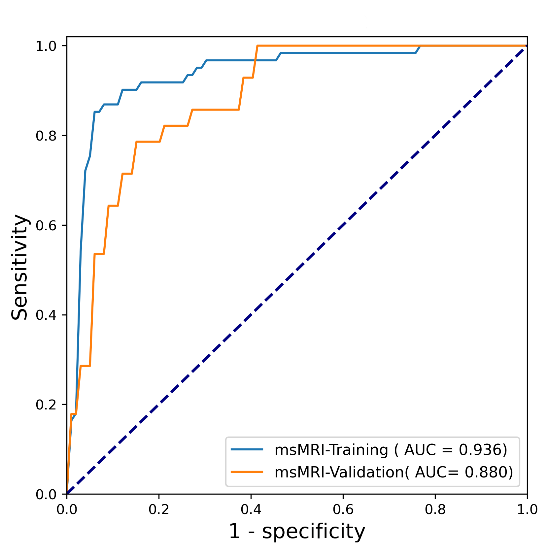
(e)**

**Fig. S3.** ROC curves of radiomics models. The training set (a) and validation set (b) for all models in Task 1. The training set (c) and validation set (d) for all models in Task 2. The training set and validation set of msMRI model (e).

**Table S3**

Delong test for ROC curves in radiomics models.

| Training set | *P* value | Validation set | *P* value |
| --- | --- | --- | --- |
| msMRI vs DCE | **< 0.001** | msMRI vs DCE | **0.001** |
| msMRI vs T2WI | **< 0.001** | msMRI vs T2WI | **0.003** |
| msMRI vs ADC | **< 0.001** | msMRI vs ADC | **0.010** |
| msMRI vs DCE + T2WI | **0.001** | msMRI vs DCE + T2WI | **0.004** |
| msMRI vs DCE + ADC | **< 0.001** | msMRI vs DCE + ADC | **0.007** |
| msMRI vs T2WI + ADC | **0.001** | msMRI vs T2WI + ADC | 0.243 |

*P* values less than 0.05 were bold.

**Table S4**

Rad-score calculation formula of radiomics model of msMRI.

| Model | Rad-score |
| --- | --- |
| msMRI | -0.43869792+(-1.0405361)*original_gldm_DependenceVariance+0.09012202*log-sigma-0-5-mm-3D_gldm_DependenceNonUniformityNormalized+0.24964679*log-sigma-2-0-mm-3D_firstorder_Median+(-0.22199674)*log-sigma-2-0-mm-3D_glszm_LowGrayLevelZoneEmphasis+(-0.34987193)*wavelet-LLH_firstorder_Skewness+(-0.98015612)*wavelet-LLH_glcm_Correlation+0.14851783*wavelet-LHL_glszm_SizeZoneNonUniformityNormalized+(-0.34575083)*wavelet-HLH_glszm_GrayLevelNonUniformityNormalized+0.3144722*wavelet-HLH_glszm_SmallAreaEmphasis+0.16718045*wavelet-HHL_glszm_SmallAreaEmphasis+(-0.75371555)*wavelet-HHH_glszm_SmallAreaHighGrayLevelEmphasis+(-0.50143147)*original_shape_Elongation+0.08239135*log-sigma-2-0-mm-3D_glszm_LargeAreaLowGrayLevelEmphasis+0.46590062*wavelet-LHL_firstorder_Mean+0.40129806*wavelet-LHL_glcm_Imc1+(-0.28796596)*wavelet-LHH_gldm_DependenceVariance+(-0.44453053)*wavelet-HHL_firstorder_Minimum+0.42458313*wavelet-HHH_glszm_SizeZoneNonUniformity+0.19617231*log-sigma-0-5-mm-3D_gldm_LargeDependenceEmphasis+0.0822812*log-sigma-1-0-mm-3D_firstorder_Kurtosis+0.34456616*log-sigma-1-5-mm-3D_glszm_SmallAreaHighGrayLevelEmphasis+0.22952234*log-sigma-2-0-mm-3D_firstorder_10Percentile+0.37510434*wavelet-LHH_glszm_LargeAreaEmphasis+0.23461566*wavelet-HLL_gldm_LargeDependenceHighGrayLevelEmphasis |
